# Comparison of the accuracy of three interproximal reduction methods used in clear aligner treatment

**DOI:** 10.1007/s00784-024-05499-4

**Published:** 2024-01-15

**Authors:** Pelinsu Güleç-Ergün, Ayça Arman-Özçırpıcı, Azize Atakan-Kocabalkan, Nilüfer İrem Tunçer

**Affiliations:** Department of Orthodontics, Faculty of Dentistry, Başkent University, Yukarıbahçelievler Mah. 82. Sokak No. 26 06490, Bahçelievler, Ankara Turkey

**Keywords:** Abrasive strips, Anxiety, Consistency, Oscillating discs, Patient perception, Stripping

## Abstract

**Objectives:**

To comparatively assess 3 interproximal reduction (IPR) methods used in clear aligner treatment with regard to accuracy, and patient perception of discomfort and anxiety.

**Materials and methods:**

A total of 42 patients, treated with the Invisalign® system, were included in this prospective trial and received one of the following IPR methods: hand-operated abrasive strips (group 1; 14 patients, 150 teeth), motor-driven 3/4 oscillating segmental discs (group 2; 14 patients, 134 teeth), or motor-driven abrasive strips (group 3; 14 patients, 133 teeth). Accuracy was evaluated using the difference between planned and executed IPR. Anxiety and discomfort levels experienced by the patients were evaluated using a questionnaire of 17 questions.

**Results:**

The accuracy of IPR was high in groups 2 and 3; however, it was low in group 1 with the executed IPR significantly less than the planned amount. On quadrant-level, executed IPR was significantly less in the upper left quadrant in group 1, and significantly more in the upper right quadrant in group 2. The difference between planned IPR and executed IPR was significant for teeth 11, 21, 32, 33, and 43 in group 1, indicating deficiency. The average difference between planned IPR and executed IPR was 0.08 mm for group 1, 0.09 mm for group 2, and 0.1 mm for group 3. Anxiety and discomfort levels did not differ between the methods, but a negative correlation was observed between age and discomfort and anxiety levels.

**Conclusions:**

The overall accuracy of the 2 motor-driven IPR methods was found to be better than the hand-operated system. Maxillary central incisors and mandibular canines were more prone to IPR deficiency when hand-operated abrasive strips were utilized. Patients were similarly comfortable with all 3 methods, and discomfort and anxiety levels decreased with age.

**Clinical relevance:**

Motor-driven methods have proven to be more effective when compared to the hand-operated ones by means of precision, speed, and patient comfort. If the clinician favors a hand-operated method, it may be advised to perform slightly more IPR especially on mandibular canines and maxillary central incisors.

**Supplementary Information:**

The online version contains supplementary material available at 10.1007/s00784-024-05499-4.

## Introduction

Technological advances in the orthodontic practice led clear aligner treatment (CAT) meet the demand for a minimally visible and customized treatment modality; however, it is still one of the main topics of scientific research of how to maximize treatment efficiency [1, 2]. Orthodontic treatment without the use of bands, brackets, and wires was first described in 1945 by Kesling [3] who used a flexible tooth positioning appliance to move the teeth. Following his philosophy, the Invisalign® system (Align Technology Inc, Santa Clara, CA, USA) took it further by using computer-aided design and computer-aided manufacturing (CAD-CAM) technology to produce a series of transparent and removable appliances [4, 5]. This new treatment approach offered the advantage of improved aesthetics, increased patient comfort, and better oral hygiene when compared to the conventional fixed mechanics [1, 6, 7]. In this system, digital scans are converted into virtual models via stereolithographic (STL) technology and processed with the ClinCheck™ software (Align Technology Inc, Santa Clara, CA, USA) to simulate virtual tooth movements and to plan where, when and how much interproximal reduction (IPR) is needed [4, 8, 9]. During the process, IPR is planned according to the clinical case requirements such as the amount of crowding, Bolton excess, molar and canine relationships, and overjet to achieve well-aligned teeth with optimal interproximal contacts [1, 4].

The success of CAT relies on various factors. These are patient-related factors such as compliance to the appliances, bone turn-over rate, and crown and root morphology of the teeth, operator-related factors such as accurate execution of the planned IPR, and an appropriate and realistic treatment plan, and mechanical factors such as the shape and position of the attachments, and material and thickness of the clear aligners [10–14]. Furthermore, the malocclusion, tooth movements required to address it, and the type of tooth to be moved are also influential on the final result.

IPR is used in the orthodontic practice to reduce the mesiodistal width of a tooth in order to resolve crowding and eliminate Bolton discrepancy, to treat black triangles by reshaping and approximating neighboring tooth, and, in the case of CAT, to provide teeth the space to carry out planned tooth movements [15–20]. Clinically, the most preferred IPR techniques include thin diamond burs or diamond-coated discs used with a handpiece, and hand- or motor-operated abrasive metal strips [15, 17]. Diamond-coated metal strips adapt easily to the proximal contours of the teeth and bend without deformation owing to its flexible nature while providing optimum tactile control and protection for the lips and cheeks [21]. Although metal strip systems are claimed to offer more precise IPR, they require longer chair-time when compared to the motor-operated systems. On the other hand, motor-operated oscillating segmental discs are designed to be one-sixth the size (60°) of a standard disc and remove the enamel by making oscillating movements with a pivot angle of 30°. Like metal strips, oscillating segmental discs are also shown to protect soft-tissues which eliminates the need for lip and cheek protectors [22].

Precise execution of IPR is considered to be crucial for proper fitting of the aligners and complete realization of digitally planned tooth movements in CAT. However, there is a lack of reliable evidence on the accuracy of different IPR methods. Therefore, the aims of this study were to compare the consistency between planned IPR and executed IPR during CAT using 3 different IPR methods; hand-operated diamond strips, motor-driven 3/4 oscillating segmental discs, and motor-driven abrasive strips, and to evaluate patient perception of discomfort and anxiety with these methods.

The null hypothesis was that there is no statistically significant difference between the planned and executed amounts of IPR, regardless of the method.

## Materials and methods

This prospective clinical study was approved by …Başkent University Institutional Review Board and Ethics Committee (Project no: D-KA 21/13) and supported by …Başkent University Research Fund. Inclusion criteria were (1) patients receiving Lite, Moderate or Comprehensive Invisalign® treatment packages (Align Technologies Inc, San Jose, CA, USA) with IPR prescription, (2) patients presenting mild to moderate crowding, and (3) full permanent dentition without impacted, missing or supernumerary teeth. Exclusion criteria were patients who (1) were candidates for extraction treatment, (2) received any dental procedure during the treatment which altered the mesiodistal width of the teeth other than IPR, (3) presented active periodontal disease, and (4) had undergone orthodontic treatment previously.

**Fig. 1:**
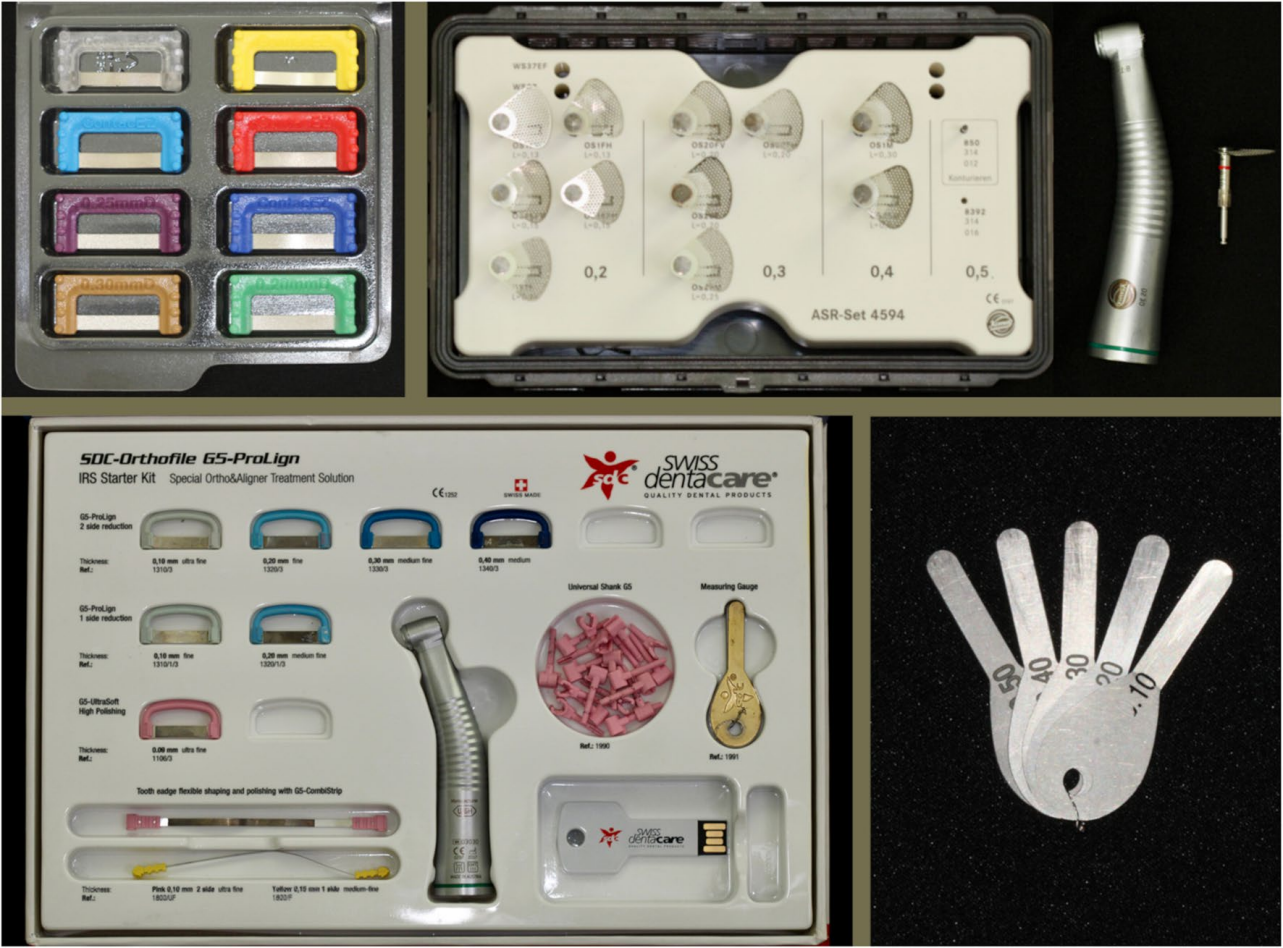
**A** Hand-operated abrasive strips; **B** motor-driven 3/4 oscillating segmental discs; **C** motor-driven abrasive strips; **D** interproximal gauge

Sample size calculation performed with 80% power to detect an outcome of 0.5 mm of difference between the planned and the executed IPR per arch, with a significance level of 0.05 and 10% potential drop-out suggested that 42 patients should be included in the study [1]. Patients were randomly assigned to one of the 3 IPR groups in the order of starting treatment. Patients in group 1 (*n* = 14, 150 teeth) received IPR with hand-operated abrasive strips (ContacEZ, Ortho Classic®, Vancouver, WA, USA), patients in group 2 (*n* = 14, 134 teeth) received IPR with motor-driven 3/4 oscillating segmental discs (KOMET, Sterisafe® A6, Rock Hill, SC, USA), and patients in group 3 (*n* = 14, 133 teeth) received IPR with motor-driven abrasive strips (SWISS, Orthofile, Swiss Dentacare®, Switzerland) (Fig. 1).

IPRs were planned to be performed when contacts were easily accessible using the ClinCheck™ software, and all IPRs were performed by the same experienced orthodontist (…A.A.Ö.) in a single center. The amount of crowding at the beginning of treatment and linear displacement between the contact points of the anterior teeth (Little’s Irregularity Index (LII)) at the session of the first IPR were calculated (MeshLab ver. 2022.02, ISTI-CNR, Rome, Italy) [23]. A metal interproximal gauge (KOMET, Rock Hill, SC, USA) was used to quantify the amount of stripping by entering the IPR site parallel to the long axes of the neighboring teeth without exerting pressure and pushing the teeth away from each other.

Scans were taken at the beginning of treatment (T0) and at the end of first set of aligners (T1) using an intraoral scanner (iTero Element 5D, Align Technologies Inc, San Jose, CA, USA). Prior to the scans, patients were asked to brush their teeth if dental plaque was evident and teeth were dried thoroughly to remove any kind of remnants which may lead to false readings.

Mesiodistal width of the teeth was noted using the arch length-tooth size discrepancy table (Bolton function) of the ClinCheck™ software at T0 and T1. It was assumed that prescribed IPR was carried out equally on either neighboring tooth. The difference between T1 and T0 gave the exact amount of executed IPR. Accuracy of IPR was assessed by calculating the absolute mean difference between planned and executed IPR, where absolute values prevented losing data. The reliability of the arch length-tooth size discrepancy table of the ClinCheck™ software was validated by using T0 and T1 widths of the teeth that were not subjected to IPR (*n* = 315). As these teeth were kept intact throughout the treatment, both measurements were expected to be the same and ICC value equal to 1.

Patients were asked to complete an anonymous questionnaire, on the session of the first IPR, to evaluate discomfort and anxiety levels induced by the IPR methods. The questionnaire statements were taken from validated questionnaire studies written in the native language of the patients [24, 25]. The finalized questionnaire consisted of 17 questions, 2 for demographic data, and 15 for the evaluation of discomfort and anxiety (Supplementary material). Of these 15 questions, 3 were yes-or-no questions, and 12 were rating scale questions on a 100-mm visual analog scale (VAS). Cutoff points for rating scale questions were determined as follows; none (0–4), mild (5–44), moderate (45–74), and severe (75–100) [26]. Questionnaire statements were validated on a representative sample of 20 before commencement of the study.

### Statistical analysis

Statistical analyses were performed using SPSS software package (version 22; IBM, Armonk, NY, USA). Shapiro–Wilk test showed that the data was non-normally distributed. Mann–Whitney *U*, Kruskal–Wallis *H*, Wilcoxon signed rank, and Chi-squared tests were used for comparisons of the data derived from the clinical step of the study, and Kruskal–Wallis *H* and Spearman’s signed rank tests were used for the results of the questionnaire.

## Results

A total of 42 patients and 417 teeth were included in the data analysis. The ICC value calculated for the reliability of the arch length-tooth size discrepancy table of the ClinCheck™ software was found to be 0.996 with good repeatability. The mean difference between T0 and T1 readings was − 0.09 mm (median, − 0.07 mm). The Cronbach’s alpha was 0.83 for the questionnaire statements.

Demographic characteristics, distribution of the malocclusion type, the amount of crowding at the beginning of treatment, and LII values at the session of the first IPR were found similar between the groups (Table 1).

**Table 1:** Demographic characteristics of the groups

	Group 1 (ContacEZ) (*n* = 14)		Group 2 (KOMET) (*n* = 14)		Group 3 (SWISS) (*n* = 14)		*p* †,‡
Variables	Median (min–max) or percent	SD	Median (min–max) or percent	SD	Median (min–max) or percent	SD	
Age (years)	31.5 (14–55)	12.36	36.5 (12–49)	10.8	36.5 (14–61)	10.3	0.552†
Sex							
Female	71%		71%		85%		0.592†
Male	29%		29%		15%		
Malocclusion type							
Class I	9		7		7		0.912†
Class II	5		7		6		
Class III					1		
Amount of crowding (mm)							
Upper arch	1.35 (0.2–4.1)	1.28	1.65 (0.5–5.2)	1.19	1.7 (0.1–3.9)	1.17	0.953‡
Lower arch	1.05 (0.2–5.3)	1.77	1.05 (0.40–7)	1.89	1.75 (0.1–4.8)	1.58	0.968‡
Contact displacement before the first IPR according to Little’s Irregularity Index							
Upper arch	1.61 (0–2.4)	1.39	1.63 (0–2.5)	1	1.58 (0–2.4)	1.34	0.727‡
Lower arch	2.26 (0–2.9)	1.52	2.35 (0–3.1)	1.57	2.32 (0–2.7)	1.74	0.995‡

*Indicates statistical significance *p* < 0.05; IPR, Interproximal reduction; Min-max, Minimum-maximum; SD, Standard Deviation

†Chi-squared test

‡Kruskal Wallis H test

Table 2 shows the comparison between planned and executed IPR within the groups. The overall average value of executed IPR was significantly less than the planned amount in group 1 (*p* = 0.003), yet similar in groups 2 (*p* = 0.511) and 3 (*p* = 0.659). On quadrant basis, executed IPR in the upper left quadrant in group 1 was significantly less than the planned amount (*p* = 0.036); however, it was significantly more in the upper right quadrant in group 2 (*p* = 0.021).

**Table 2:** Comparison of planned IPR (mm) and executed IPR (mm) within groups

Measurement	Group 1 (ContacEZ)				Group 2 (KOMET)				Group 3 (SWISS)			
	*n*	Planned	Executed	*p*†	*n*	Planned	Executed	*p*†	*n*	Planned	Executed	*p*†
		Median (min–max)	Median (min–max)			Median (min–max)	Median (min–max)			Median (min–max)	Median (min–max)	
Quadrant												
Upper right	28	0.24 (0.1–0.4)	0.2 (0–0.44)	0.070	24	0.2 (0.1–0.3)	0.28 (0–0.91)	0.021*	25	0.2 (0.1–0.5)	0.19 (0.01–0.54)	0.819
Upper left	34	0.26 (0.1–0.5)	0.22 (0–0.55)	0.036*	23	0.23 (0.1–0.5)	0.22 (0–0.47)	0.581	26	0.24 (0.1–0.45)	0.27 (0.04–0.8)	0.761
Lower right	44	0.21 (0.1–0.3)	0.2 (0–0.61)	0.148	41	0.23 (0.1–0.5)	0.23 (0–0.56)	0.494	38	0.28 (0.1–0.5)	0.32 (0.04–1)	0.713
Lower left	44	0.2 (0.1–0.3)	0.18 (0–0.5)	0.437	46	0.21 (0.1–0.5)	0.2 (0–0.7)	0.433	44	0.3 (0.1–0.5)	0.34 (0.02–0.96)	0.599
Tooth number												
11	9	0.27 (0.2–0.4)	0.18 (0–0.41)	0.110	10	0.2 (0.1–0.3)	0.3 (0–0.91)	0.139	7	0.2 (0.1–0.4)	0.25 (0.07–0.42)	0.125
12	9	0.22 (0.1–0.4)	0.2 (0.09–0.44)	0.593	7	0.21 (0.1–0.3)	0.23 (0.09–0.32)	0.553	8	0.19 (0.2–0.4)	0.28 (0.25–0.54)	0.233
13	7	0.24 (0.1–0.4)	0.21 (0.02–0.34)	0.125	6	0.15 (0.1–0.25)	0.22 (0.07–0.29)	0.092	6	0.17 (0.1–0.25)	0.08 (0.01–0.18)	0.026*
21	10	0.33 (0.1–0.4)	0.24 (0–0.34)	0.093	10	0.23 (0.1–0.4)	0.22 (0–0.42)	0.799	7	0.23 (0.1–0.4)	0.31 (0.07–0.8)	1
22	10	0.32 (0.1–0.5)	0.28 (0.02–0.47)	0.031*	8	0.24 (0.1–0.5)	0.27 (0.02–0.47)	0.307	5	0.21 (0.1–0.45)	0.16 (0.12–0.62)	0.501
23	9	0.22 (0.1–0.5)	0.21 (0.09–0.55)	0.889	5	0.17 (0.1–0.4)	0.13 (0.09–0.22)	0.501	7	0.24 (0.1–0.45)	0.21 (0.04–0.4)	0.125
31	13	0.23 (0.1–0.3)	0.23 (0.08–0.43)	0.807	13	0.29 (0.1–0.5)	0.31 (0.09–0.56)	0.289	12	0.3 (0.2–0.5)	0.31 (0.06–0.74)	0.969
32	12	0.2 (0.1–0.3)	0.2 (0.04–0.5)	0.753	13	0.28 (0.1–0.5)	0.29 (0.06–0.57)	1	12	0.32 (0.1–0.5)	0.42 (0.07–0.96)	0.476
33	11	0.2 (0.1–0.3)	0.15 (0–0.34)	0.197	12	0.22 (0.1–0.4)	0.12 (0–0.37)	0.021*	11	0.3 (0.1–0.5)	0.36 (0.02–0.93)	0.722
34	8	0.1 (0.1–0.15)	0.12 (0.02–0.34)	1	7	0.15 (0.1–0.4)	0.1 (0.01–0.36)	0.091	8	0.2 (0.1–0.25)	0.21 (0.1–0.58)	0.344
41	13	0.23 (0.1–0.3)	0.2 (0–0.32)	0.209	12	0.28 (0.15–0.5)	0.27 (0–0.5)	0.635	11	0.31 (0.2–0.5)	0.35 (0.04–0.57)	0.398
42	11	0.24 (0.2–0.3)	0.24 (0.1–0.52)	0.645	10	0.3 (0.2–0.45)	0.35 (0.1–0.52)	0.213	11	0.3 (0.1–0.5)	0.34 (0.11–1)	0.441
43	11	0.22 (0.1–0.3)	0.15 (0.01–0.25)	0.074	11	0.2 (0.1–0.4)	0.14 (0.01–0.32)	0.068	9	0.29 (0.1–0.4)	0.29 (0.06–0.69)	0.362
44	8	0.13 (0.1–0.3)	0.20 (0.03–0.61)	0.233	7	0.15 (0.1–0.4)	0.21 (0.1–0.56)	0.027*	6	0.17 (0.15–0.25)	0.26 (0.06–1)	0.674
Overall	150	0.22 (0.1–0.5)	0.2 (0–0.61)	0.003*	134	0.22 (0.1–0.5)	0.23 (0–0.91)	0.511	133	0.26 (0.1–0.5)	0.29 (0.01–1)	0.659

*Statistical significance *p* < 0.05; *Min–max*, minimum–maximum

†Wilcoxon signed rank test

Table 3 presents the comparison of accuracy of the IPR methods by means of the average difference between planned and executed IPR. According to this, the accuracy of the IPR method in group 1 was significantly low for teeth 11, 21, 32, 33, and 43 (*p* = 0.021). Table 4 presents the accuracy of IPR between the arches (upper/lower), sides (left/right), and groups of teeth (incisors/canines/premolars), where no significant difference was evident.

**Table 3:** Comparison of the accuracy of execution of planned IPR (mm) within and between the groups

Measurement	Group 1 (ContacEZ)			Group 2 (KOMET)			Group 3 (SWISS)			Between groups
	*n*	Median (min–max)	*p*‡	*n*	Median (min–max)	*p*‡	*n*	Median (min–max)	*p*‡	*p*‡
Quadrant										
Upper right	28	0.08 (0.01–0.29)	0.756	24	0.1 (0.01–0.76)	0.877	25	0.09 (0–0.36)	0.362	0.816
Upper left	34	0.07 (0–0.27)		23	0.08 (0–0.22)		26	0.07 (0–0.7)		0.102
Lower right	44	0.06 (0–0.46)		41	0.09 (0–0.26)		38	0.11 (0–0.85)		0.629
Lower left	44	0.07 (0.01–0.22)		46	0.09 (0–0.5)		44	0.1 (0–0.76)		0.726
Tooth number										
11	9	0.13 (0.01–0.29)	0.021*	10	0.17 (0.01–0.76)	0.626	7	0.09 (0.02–0.27)	0.758	0.788
12	9	0.08 (0.01–0.12)		7	0.04 (0.01–0.12)		2	0.08 (0–0.16)		–
13	7	0.08 (0.01–0.09)		6	0.09 (0.03–0.14)		6	0.07 (0.02–0.2)		0.315
21	10	0.15 (0.02–0.27)		10	0.10 (0.02–0.21)		7	0.11 (0–0.7)		0.144
22	10	0.04 (0–0.16)		8	0.06 (0–0.18)		4	0.07 (0.02–0.11)		–
23	9	0.07 (0–0.22)		5	0.1 (0.02–0.11)		5	0.03 (0.02–0.2)		0.302
31	13	0.04 (0.01–0.13)		13	0.08 (0–0.19)		12	0.1 (0.01–0.44)		0.491
32	12	0.11 (0.04–0.2)		13	0.11 (0–0.23)		12	0.11 (0–0.76)		0.164
33	11	0.08 (0.02–0.22)		12	0.09 (0–0.3)		11	0.12 (0.02–0.63)		0.557
34	8	0.04 (0.01–0.19)		7	0.07 (0.03–0.14)		8	0.07 (0–0.43)		0.288
41	13	0.06 (0–0.2)		12	0.06 (0–0.24)		11	0.1 (0.03–0.3)		0.140
42	11	0.05 (0–0.22)		10	0.11 (0–0.22)		11	0.1 (0–0.7)		0.561
43	11	0.11 (0–0.26)		11	0.1 (0.02–0.26)		9	0.12 (0–0.59)		0.920
44	8	0.08 (0–0.46)		7	0.06 (0–0.16)		6	0.15 (0.03–0.85)		0.550
Overall	150	0.08 (0–0.46)		134	0.09 (0–0.76)		133	0.1 (0–0.85)		0.436

*Statistical significance *p* < 0.05; *Min–max*, minimum–maximum

‡Kruskal–Wallis *H* test

**Table 4:** Comparison of the accuracy of execution of planned IPR (mm) according to arch, side, and group of teeth

	*n*	Median (min–max)	*p*
Arch			
Upper	160	0.75 (0–0.76)	0.956†
Lower	257	0.7 (0–0.85)	
Side			
Left	217	0.7 (0–0.76)	0.922†
Right	200	0.8 (0–0.85)	
Group of teeth			
Incisors	236	0.7 (0–0.76)	0.527‡
Canines	103	0.8 (0–0.63)	
Premolars	72	0.7 (0–0.85)	

*Statistical significance *p* < 0.05; *Min–max*, minimum–maximum

†Mann–Whitney *U* test

‡Kruskal–Wallis *H* test

The results of the questionnaire showed that patients’ discomfort and anxiety levels were similar between the groups regardless of the IPR method; however, scores in group 1 were slightly higher than the other 2 groups in general. Patients who were priorly informed about the procedure were less likely to make research (Table 5). Moreover, correlation tests revealed that a negative correlation existed between age, and the severities of pain (*r* =  − 0.548, *p* = 0.042) and tooth sensitivity (*r* =  − 0.540, *p* = 0.046).

**Table 5:** Summary of the questionnaire results

Question	Group 1 (ContacEZ)		Group 2 (KOMET)		Group 3 (SWISS)		Between groups
	Scale rating or %	SD	Scale rating or %	SD	Scale rating or %	SD	*p*‡
How worried are you regarding the procedure which is about to be performed?	3.71	2.89	3.5	3.16	3.71	2.81	0.818
How worried are you right now?	2.57	2.74	2.21	2.97	1.79	2.81	0.779
Would you still be worried if you knew at the beginning of the treatment that this procedure was going to be performed?							
Yes/no	50/50		38.5/61.5		42.9/57.1		0.747
How would you rate the severity of the discomfort on your cheeks?	2.5	2.65	1.14	1.88	0.93	1.82	0.17
How would you rate the duration of the discomfort on your cheeks?	1.93	2.46	1.21	2.12	0.86	1.66	0.516
How would you rate the severity of the pain you felt during the procedure?	2.93	3.08	2.43	2.93	2.86	2.25	0.812
How would you rate the duration of the pain you felt during the procedure?	2.36	2.27	2.21	2.78	2.5	2.1	0.737
How would you rate the severity of the discomfort on your gums?	3.07	3.08	2.64	2.71	2.5	2.35	0.958
How would you rate the duration of the discomfort in your gums?	2.93	2.95	2.36	2.5	2.57	2.5	0.943
How would you rate the severity of tooth sensitivity?	3.36	3.67	2.21	2.49	2.93	2.02	0.528
How would you rate the duration of tooth sensitivity?	2.64	2.53	2.36	2.82	2.93	2.02	0.685
How would you rate the bleeding on your gums during the procedure?	2.98	2.34	2.77	2.52	3.03	2.97	0.844
How would you rate the amount of gingival bleeding?	2.23	2.12	2.44	2.35	2.69	2.31	0.732
Did you know at the beginning of the treatment that this procedure was going be performed?							
Yes/no	57.1/42.9		42.9/57.1		71.4 / 28.6		0.135
Have you done any research on this procedure?							
Yes/no	37.5/62.5		66.7/33.3		0 / 100		0.311

‡Kruskal–Wallis *H* test

## Discussion

IPR is an essential component of CAT which creates room for teeth to perform digitally planned movements and has to be executed precisely in order to achieve the desired outcome. Therefore, the primary aim of this study was to investigate the consistency between planned and executed IPR for 3 IPR methods including hand-operated abrasive diamond strip system, motor-driven oscillating segmental discs, and motor-driven abrasive strips, in patients receiving CAT with the Invisalign® system. The secondary aim was to assess discomfort and anxiety levels induced by these methods. This study stands out among the others in the literature with regard to its in vivo nature and prospective design which made standardization of the interventions possible. Furthermore, all IPRs were executed by an experienced orthodontist to minimize the risk of uncontrolled IPR, especially with the motor-driven methods that would lead to over-stripping and distortion of the contact surface morphology. This is also the first study to assess patients’ perceptions and experiences with different IPR methods during CAT.

The null hypothesis is rejected. The overall executed IPR was less than the planned amount with the hand-operated abrasive strip system (ContacEZ), yet similar with the motor-driven systems (KOMET and SWISS), even though the amount of contact displacement was similar between the groups prior to the first IPR. These findings are in line with De Felice et al.’s [1] who showed that manual strip system fell short to fully execute the planned IPR. Although this system is claimed to provide a more reliable and precise stripping process due to the flexibility of the strips, they have to be used incrementally from thinner to thicker strips, and the clinician needs to enter the same region iteratively with a significant pressure [8, 16, 17]. This may lead to displacement of the tooth in its socket and lead to a false reading on the interproximal gauge. Furthermore, it is clinically more tiring and time consuming, especially when a marked amount of IPR is planned, which may give the clinician a false impression that the targeted amount is reached. On the contrary, motor-driven IPR methods are shown to provide more effective stripping than hand-operated methods by means of speed and the need for less amount of pressure entering the contact points [27–29].

Executed IPR was significantly deficient on the mandibular canines with the hand-operated abrasive strip system, which is in line with the findings of Kalemaj and Levrini [16], because canines are in tight contact with the adjacent teeth and are frequently pushed out of the dental arch in the case of crowding, which makes it challenging to precisely implement the planned IPR [30]. Furthermore, mandibular teeth are usually in a more crowded state than the maxillary teeth, which is supported by the findings of the present study showing that IPR was dominantly carried out on the mandibular arch (61.6%) when compared to the maxillary arch (38.3%) that was also documented by Hariharan et al. [31].

Executed IPR exceeded the planned amount on the upper right quadrant with motor-driven oscillating segmental discs and could not reach the planned amount on the upper left quadrant with the hand-operated abrasive strip system; however, the overall amount of executed IPR was similar between upper and lower arches, as well as left and right quadrants when the sample was evaluated regardless of the IPR system.

Johner et al. [14] evaluated the accuracy of 3 different IPR methods (hand-operated abrasive strips, motor-driven oscillating discs, and motor-driven abrasive strips) on extracted premolar teeth with an in vitro study design. They showed that the amount of IPR was generally less than expected in all 3 groups. Although their results are in line with the abrasive strip group in the present study, their motor-driven IPR groups present conflicting results with ours. These differences seem to arise from the study designs, one being conducted in an actual biological setting with teeth surrounded by its periodontal ligament, differing in crown morphology and in a crowded state, and the other in an artificial setting with only premolar teeth mounted in silicone to mimic the periodontal ligament.

Based on the findings of this study, motor-driven methods have proven to be more effective when compared to the hand-operated ones by means of precision, speed, and patient comfort. However, if the clinician favors a hand-operated method, it may be advised to perform slightly more IPR especially on mandibular canines and maxillary central incisors. Also, a brief preliminary alignment phase grants easy access to the contact surfaces which is believed to increase the accuracy of IPR with less damage to the tooth shape and contact surface morphology. Overall, a precisely executed IPR in clear aligner treatment is the way to complete realization of the planned tooth movements and ultimately to a successful treatment.

According to the results of the questionnaire, patients were mildly anxious about IPR before the procedure, and their anxiety levels decreased after. A negative correlation existed between age and anxiety and discomfort levels. On the other hand, patients reported similar levels of pain and discomfort with both hand-operated and motor-driven IPR methods, which was unexpected because motor-driven IPR is thought to be more perturbative for the patients. Considering that motor-driven methods provide more accurate IPR, and that patients’ anxiety and discomfort levels are similar with the hand-operated method, motor-driven methods can be preferred over hand-operated methods.

Today, websites and social media platforms are frequently referred by the patients for healthcare research. However, the lack of regulations and the fact that any individual can upload a content pose the risk of misinformation [32]. Results of the questionnaire showed that patients who were priorly informed about the IPR procedure tended to make less research. This means that patients can be kept away from the information pollution on the internet and the treatment can be led by the orthodontist in a more reliable environment.

## Limitations

Although the arch length-tooth size discrepancy table of the ClinCheck™ software is claimed to be prone to measurement errors, its repeatability was found to be very good which shows that the quantitative uncertainty was very low. There is some margin of error in all measurement methods (manual or digital), but the system can be accepted as reliable as long as this does not significantly alter the expected effect [16, 33, 34]. Blindness, on the other hand, is another favorable feature of the software.

The demonstrated differences between the IPR methods are statistically significant; however, they may not be indicative of clinical significance.

## Conclusions

- The overall results of this in vivo study showed a discrepancy between planned and executed IPR with the hand-operated abrasive strip system, tending to produce less enamel reduction than planned.
- The hand-operated abrasive strips fell short to fully realize the planned amount of IPR, especially on the maxillary central incisors and mandibular canines.
- The consistency of IPR with motor-driven oscillating segmental discs and motor-driven abrasive strips was high.
- Although the difference between the hand-operated and motor-driven methods was statistically significant, it may not be clinically relevant.
- According to the results of the questionnaire, both motor-driven and hand-operated methods caused similar levels of discomfort.

### Supplementary Information

Below is the link to the electronic supplementary material.

Supplementary file1 (DOCX 111 KB)

## Data Availability

Data availability is upon request to the corresponding author.
