# Supplementary material for: Comparison of the accuracy of three interproximal reduction methods used in clear aligner treatment
Source: Clin Oral Investig. 2024 Jan 15;28(1):95. doi: 10.1007/s00784-024-05499-4 (PMC10788318; doi:10.1007/s00784-024-05499-4)
Supplement: Supplementary file 1 — Supplementary file1 (DOCX 111 KB) [file 784_2024_5499_MOESM1_ESM.docx]

**… University Faculty of Dentistry Department of Orthodontics**

**Patient Comfort Questionnaire for Interproximal Reduction Procedure**

This questionnaire intends to evaluate your experience with the interproximal reduction procedure which will be performed as a part of your clear aligner treatment. Your answers and comments will remain confidential and will be used only for the purpose of research and statistics. When answering the questions, please mark the score that you think is most suitable for you from 0 to 10. Thank you in advance for your interest and help.

1. Age:
2. Sex:

1. How worried are you regarding the procedure which is about to be performed?

none severe

Answer the following questions after the interproximal reduction procedure.

1. How worried are you right now?

none severe

1. Would you still be worried if you knew at the beginning of the treatment that this procedure was going to be performed?

Yes / No

1. How would you rate the severity of the discomfort on your cheeks?

none severe

1. How would you rate the duration of the discomfort on your cheeks?

none extended

1. How would you rate the severity of the pain you felt during the procedure?

none severe

1. How would you rate the duration of the pain you felt during the procedure?

none extended

1. How would you rate the severity of the discomfort on your gums?

none severe

1. How would you rate the duration of the discomfort in your gums?

none extended

1. How would you rate the severity of tooth sensitivity?

none severe

1. How would you rate the duration of tooth sensitivity?

none extended

1. How would you rate the bleeding on your gums during the procedure?

none extended

1. How would you rate the amount of gingival bleeding?

none severe

Yes / No

1. Did you know at the beginning of the treatment that this procedure was going be performed?

Yes / No

1. Have you done any research on this procedure?

Thank you for completing our questionnaire!
